# Medical Students’ Perception of Educational Environment in a Newly Established Institute of National Importance in Eastern Uttar Pradesh: A Cross-Sectional, Questionnaire-Based Study

**DOI:** 10.7759/cureus.85092

**Published:** 2025-05-30

**Authors:** Vijaya Laxmi, Tejas K Patel, Shahid Malik, Amit Ranjan

**Affiliations:** 1 Pharmacology and Therapeutics, All India Institute of Medical Sciences, Gorakhpur, Gorakhpur, IND; 2 Medical Biochemistry, All India Institute of Medical Sciences, Gorakhpur, Gorakhpur, IND; 3 Physical Medicine and Rehabilitation, All India Institute of Medical Sciences, Gorakhpur, Gorakhpur, IND

**Keywords:** dreem questionnaire, educational environment, medical education, medical students, self-perception

## Abstract

Background

The educational environment in medical education is essential, as both physical and psychological factors significantly impact students’ learning experiences, well-being, and growth as medical professionals. A well-organized and supportive environment enhances student engagement, motivation, and academic performance. Assessing and enhancing the educational environment is crucial for ensuring its effectiveness. Students’ perceptions play a vital role in evaluating the learning atmosphere and its impact. The Dundee Ready Education Environment Measure (DREEM) is a well-established tool for assessing the educational environment. Collecting baseline data on students’ perceptions in a newly established medical college can provide valuable insights, serving as a foundation for implementing improvements and necessary changes to enhance the learning experience.

Methodology

A cross-sectional study was conducted among 412 medical students from all semesters (first to ninth semester). Students’ perceptions were assessed using the 50-item DREEM questionnaire, which covers five key domains, namely, students’ perception of learning (SPL), students’ perception of teachers (SPT), students’ academic self-perceptions (SASP), students’ perception of atmosphere (SPA), and students’ social self-perceptions (SSSP). Responses were recorded on a five-point Likert scale, and the data were analyzed using appropriate statistical methods, with a significance level set at a p-value <0.05.

Results

The mean DREEM score and the mean domain scores were calculated. The mean global DREEM score was 114.77 ± 5.40, indicating a generally positive perception of the educational environment. The total score for the SPL domain was 28.74 out of 48 (60%), reflecting a predominantly positive perception. The SPT domain scored 24.72 out of 44 (56%), suggesting progress in the right direction. The SASP domain had a total score of 17.95 out of 32 (56%), indicating a leaning toward a positive outlook. The SPA domain scored 26.9 out of 48 (56%), signifying a favorable atmosphere, while the SSSP domain had a score of 15.96 out of 28 (57%), interpreted as not too bad.

Conclusions

All domain scores exceeded 55%, and the overall total score of 114.77 on the DREEM scale confirmed a predominantly positive perception of the educational environment.

## Introduction

Gaining an insight into students’ perceptions of the educational environment is essential for assessing the effectiveness and acceptability of the current curriculum. This understanding helps create a more supportive learning atmosphere and ensures the optimal utilization of educational resources. Regular feedback and evaluation are key in recognizing and addressing gaps within the educational environment, fostering continuous improvement.

The success of undergraduate medical education is heavily influenced by the educational environment, encompassing all activities within the classroom, department, or university. The educational environment is composed of the three components of the physical environment, as well as the emotional and intellectual climates [1].

The medical curriculum is wide-ranging and highly challenging to medical students, as a large amount of information needs to be learned within a specified period, which places an enormous academic burden on the students [2]. Globally, medical educators are striving to reshape the educational environment, aiming to create a student-friendly atmosphere without compromising on standards and the quality of learning. A positive and healthy environment is crucial for the success of an effective academic curriculum. The competence of healthcare professionals plays a crucial role in patient safety and well-being, highlighting the importance of their education in supporting health initiatives.

Educational environment consists of diverse social, cultural, and moral values, and the educational experience in a medical institution has the potential to induce a lasting transformation in students’ knowledge, attitudes, skills, and behavior. Hence, evaluating educational environments has been recognized as a crucial tool for providing high-quality education. Several tools have been created to assess the environments in medical education. Various authorities, including the World Federation for Medical Education, prioritize evaluating the educational environment as a crucial aspect of medical education programs [3].

Several tools are available to assess students’ perception of the learning environment. The Dundee Ready Educational Environment Measure (DREEM) questionnaire is one of the most extensively used tools and has been developed and validated to be used on a culturally diverse student population [4]. It has high internal consistency, ranging between 89% and 91%. Other tools include the Learning Environment Assessment, Accreditation Council for Graduate Medical Education, Medical School Environment Questionnaire, and Johns Hopkins Learning Environment Scale [5].

The DREEM is a 50-item measure with a maximum score of 200 and measures the following five separate domains of the education environment: students’ perceptions of learning (SPL), students’ perceptions of teachers (SPT), students’ perceptions of atmosphere (SPA), students’ academic self-perception (SASP), and students’ social self-perceptions (SSSP).

This study aimed to gather baseline information about students’ perceptions of the educational environment in a newly established medical college using the 50-item DREEM questionnaire.

## Materials and methods

Study design, population, and sampling

This was a cross-sectional, questionnaire-based study. The study was conducted among all nine semesters of medical undergraduate students studying in the All India Institute of Medical Sciences, Gorakhpur, for more than three months. The MBBS program at our institute spans nine semesters. The structure is as follows: (a) the first professional year comprises two semesters, first and second; (b) the second professional year includes three semesters, third, fourth, and fifth; (c) the third professional year consists of four semesters, sixth, seventh, eighth, and ninth. At the time of conducting the study, all semesters were included. Students are typically enrolled in either the even-numbered or odd-numbered semesters at any given time, with one or two semesters awaiting examinations.

Students participated in this study anonymously. The questionnaire did not bear any direct identifiers and only contained details such as the semester and gender of the participant. Permission from the institutional ethics committee was obtained before conducting the study.

Questionnaire

The DREEM questionnaire was used as a measure of students’ perceptions about the educational environment. DREEM is a thoroughly validated instrument initially created to assess the educational atmosphere, tailored particularly for medical schools and other healthcare professions [6]. It comprises a 50-item inventory containing statements pertinent to the educational environment, with a maximum score of 200. It encompasses the following five domains: (1) SPT: 11 items; maximum score, 44; (2) SPL: 12 items; maximum score, 48; (3) SASP: 8 items; maximum score, 32; (4) SPA: 12 items; maximum score, 48; (5) SSSP: 7 items; maximum score, 28.

The scoring system used for the DREEM questionnaire was proposed by McAleer and Roff [7]. Each of the 50 items in the DREEM questionnaire was scored on a scale of 0 to 4, with the following assignments: 0 (strongly disagree), 1 (disagree), 2 (uncertain), 3 (agree), and 4 (strongly agree). Reverse scoring was used for the nine negative items (4, 8, 9, 17, 25, 35, 39, 48, and 50), where strongly agree was scored 0 and strongly disagree was scored 4.

Questionnaire administration

The questionnaire was administered using a paper-based format. It was distributed to participants during scheduled sessions in designated study areas. A member of the research team was present to distribute the questionnaires, provide standardized instructions, and address any questions or requests for clarification without influencing participants’ responses. Participants were informed that participation was voluntary and that they could withdraw at any time without penalty. The estimated time to complete the questionnaire was approximately 30-40 minutes. A participant information sheet outlining the objectives of the study and other relevant details, such as the voluntary nature of participation, data confidentiality, and contact information for the research team, was provided to all participants before data collection.

Data analysis

The scores were interpreted in accordance with the practical guidelines given by McAleer and Roff [7,8], as depicted in Table 1 and Table 2.

**Table 1:** Interpretation of DREEM subscales. DREEM: Dundee Ready Education Environment Measure

DREEM subscales	Interpretation of scores
Students’ perception of learning	0–12, very poor
	13–24, teaching is viewed negatively
	25–36, a more positive approach
	37–48, teaching is highly thought of
Students’ perception of teachers	0–11, abysmal
	12–22, in need of some retraining
	23–33, moving in the right direction
	34–44, model teachers
Students’ academic self-perceptions	0–8, feeling of total failure
	9–16, many negative aspects
	17–24, feeling more on the positive side
	25–32, confident
Students’ perception of atmosphere	0–12, a terrible environment
	13–24, there are many issues that need changing
	25–36, a more positive atmosphere
	37–48, a good feeling overall
Students’ social self-perceptions	0–7, miserable
	8–14, not a nice place
	15–21, not too bad
	22–28, very good socially

**Table 2:** Interpretation of overall score.

Overall score	Interpretation
0–50	Very poor
50–100	Plenty of problems
100–150	More positive than negative
150–200	Excellent

Statistical analysis

The data collected was entered into an MS Office Excel spreadsheet (Microsoft Corp., Redmond, WA, USA). Total scores and domain-specific scores for students across all semesters were calculated. The DREEM scores were presented in both continuous and categorical formats, adhering to the DREEM analysis guide (four-point Likert scale). Continuous variables were expressed as means and standard deviations, while categorical variables were reported as frequencies and percentages. The Shapiro-Wilk normality test for the data yielded significant results (p < 0.05) for all semesters and all subscale domains and the total DREEM score. This indicates that the data was not normally distributed; therefore, non-parametric tests were applied to the dataset. The Kruskal-Wallis test was performed to analyze variations in the total mean scores across five different semesters and to compare the five subscale scores between semesters. Comparisons of the mean scores between male and female students were conducted, and the Mann-Whitney U test was used to identify statistically significant differences, with a p-value <0.05 considered statistically significant. A post-hoc pairwise comparison between semesters was performed using the Dunn-Bonferroni Test, with the Bonferroni correction for increasing specificity of the test.

Ethical considerations

The study protocol was approved by the Institutional Human Ethics Committee, All India Institute of Medical Sciences, Gorakhpur (approval number: IHEC/AIIMS-GKP/BMR/158/2023), and all procedures adhered to ethical guidelines for research involving human participants. To ensure anonymity, no identifying information was collected, and completed questionnaires were stored securely and accessed only by authorized research personnel.

## Results

The data were obtained from MBBS students in their first, third, fourth, seventh, and ninth semesters. A total of 412 students filled out the questionnaire. There were 276 (67%) male and 136 (33%) female participants. Table 3 shows the demographic details of the participants.

**Table 3:** Demographic details of the study participants.

Variable	First semester	Third semester	Fourth semester	Seventh semester	Ninth semester
Gender					
Male	80 (72%)	63 (60%)	61 (67%)	41 (70%)	31 (64%)
Female	31 (28%)	41 (40%)	30 (33%)	17 (30%)	17 (30%)

**Figure 1:**
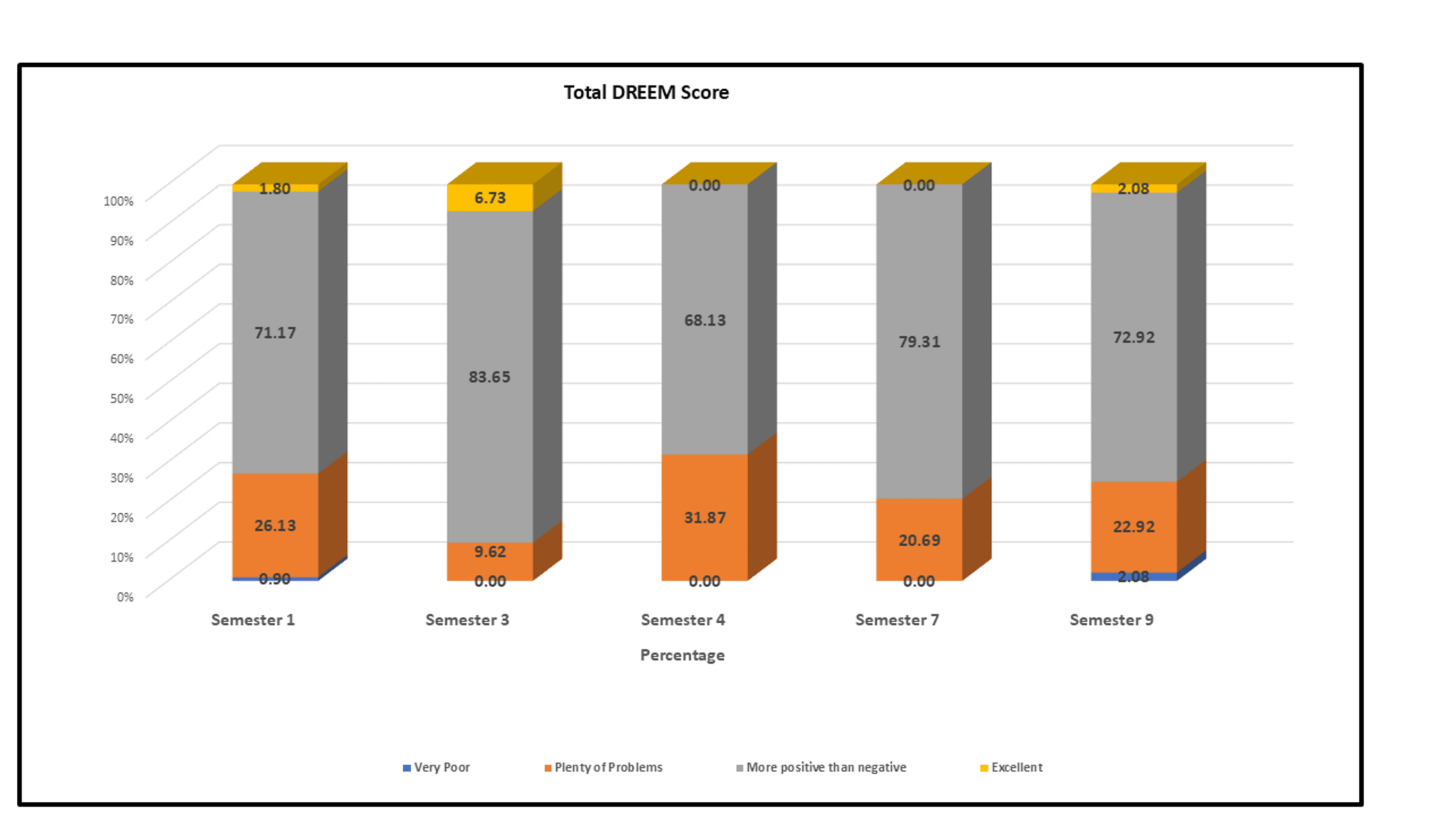
Total DREEM score across semesters. The overall DREEM score was 114.65 ± 20.05 out of 200. DREEM: Dundee Ready Education Environment Measure

The overall DREEM score in our study was 114.65 ± 20.05 out of 200. The total DREEM score across semesters is depicted in Figure 1.

The DREEM domains with total and individual scores are presented in Table 4.

**Table 4:** DREEM domains with total and individual scores. DREEM: Dundee Ready Education Environment Measure; SPL: students’ perceptions of learning; SPT: students’ perceptions of teachers; SASP: students’ academic self-perceptions; SPA: students’ perceptions of atmosphere; SSSP: students’ social self-perceptions

Domain	Interpretation	Count	Percentage
SPA: 12 items; maximum score, 48	A terrible environment	13	3.15
	There are many issues that need changing	163	39.56
	A more positive atmosphere	228	55.33
	A good feeling overall	8	2.0
SSSP: 7 items; maximum score, 28	Miserable	11	2.67
	Not a nice place	166	40.29
	Not too bad	224	54.36
	Very good socially	11	2.66
SASP: 8 items; maximum score, 32	Feeling of total failure	12	3.0
	Many negative aspects	107	27.0
	Feeling more on the positive side	251	61.0
	Confident	42	10.20
SPT: 11 items; maximum score, 44	Abysmal	0	0
	In need of some retraining	47	11.41
	Moving in the right direction	344	83.49
	Model teachers	20	4.85
SPL: 12 items; maximum score, 48	Very poor	6	1.45
	Teaching is viewed negatively	91	22.0
	A more positive approach	280	68.0
	Teaching is highly thought of	34	8.25

Table 5 presents the DREEM subscale scores of the study sample, along with a semester-wise comparison of the results. The means of all of the subscales were greater than 55%.

**Table 5:** DREEM subscale scores of the study sample, along with a semester-wise comparison of the results. The means of all the subscales were greater than 55%. Data are presented as mean ± standard deviation. One-way analysis of variance followed by post-hoc Tukey-Kramer multiple comparisons test was applied. Statistical significance is presented as ^a^: p < 0.05, ^b^: p < 0.01, and ^c^: p < 0.001. DREEM: Dundee Ready Education Environment Measure; SPL: students’ perceptions of learning; SPT: students’ perceptions of teachers; SASP: students’ academic self-perceptions; SPA: students’ perceptions of atmosphere; SSSP: students’ social self-perceptions

DREEM domain (maximum score)	First semester	Third semester	Fourth semester	Seventh semester	Ninth semester	Significant difference between domain scores of semesters
SPA (48)	24.47 ± 5.79	26.82 ± 6.56	22.73 ± 5.92	25.5 ± 5.55	25.48 ± 6.23	1:3^a^, 3:4^b^
SSSP(28)	15.11 ± 3.59	15.59 ± 3.62	14.83 ± 2.72	13.91 ± 3.35	15.54 ± 3.86	Non-significant
SASP(32)	17.36 ± 4.75	21.05 ± 4.40	17.69 ± 4.36	19.44 ± 3.59	18.52 ± 5.48	1:3^c^, 3:4^c^, 3:9^a^
SPT(44)	27.3 ± 3.68	27.82 ± 3.45	26.51 ± 4.27	26.81 ± 3.15	27.02 ± 5.40	Non-significant
SPL(48)	27.92 ± 6.39	32.21 ± 4.69	26.72 ± 6.07	28.1 ± 5.46	27.81 ± 6.81	1:3^c^,3:4, 3^c^:7, 3:9^c^

## Discussion

With the recent emphasis on enhancing quality assessment and monitoring in medical education, there has been increasing interest in strengthening student-centered teaching and learning methods. As a result, students’ perceptions of their educational environment have gained significant relevance in evaluating and improving learning experiences.

Evaluating and enhancing the educational environment is crucial for ensuring its effectiveness, relevance, and alignment with students’ learning needs. Regular assessment helps identify strengths and areas for improvement, leading to better teaching strategies, improved student outcomes, and higher satisfaction. Additionally, continuous enhancement ensures adaptability to evolving educational standards and societal demands, ultimately fostering a more effective and dynamic learning environment.

The present study was undertaken to assess the educational environment among medical undergraduate (MBBS) students at AIIMS Gorakhpur. Student feedback was utilized to test three hypotheses, namely, (i) students’ perception of the educational environment remains consistent across different semesters; (ii) domain-specific DREEM scores do not show significant variation across semesters; and (iii) there is no significant difference in students’ perception of the educational environment based on gender.

Students’ perception across semesters

The total DREEM score was the highest among students in the third semester (122 ± 19.5), followed by the ninth semester (114.37 ± 24.37), seventh semester (113.77 ± 15.76), first semester (112.52 ± 19.96), and fourth semester (108.48 ± 17.82). There was a statistically significant difference in students’ perception of the educational environment between the first and third semester (p < 0.01), between the third and fourth semester (p < 0.001), and between the third and seventh semester (p < 0.05).

The mean DREEM score of total perception (114.65 ± 20.05) indicated the overall positive view of the students toward the clinical educational environment. The total mean score obtained in this study was lower than the scores reported in similar studies. On a national level, this value is lower than the results found in previous research conducted in medical and dental schools. Internationally, our overall DREEM score was lower compared to the scores achieved in similar studies [9-12].

Comparisons between students from different semesters showed that the global DREEM score, along with the domain scores, were significantly more favorable for third-semester students compared to those in other semesters. Our findings are consistent with those of Kohli et al. [13] and Pai et al. [14].

The finding that third-semester medical students in India may have a more positive perception of the educational environment compared to other semesters can be attributed to the fact that this phase marks the shift from preclinical to paraclinical subjects, bringing them closer to clinical practice, which can be exciting and motivating. Moreover, compared to the intense preclinical years (first and second semesters) and the heavier clinical workload in later semesters, the third semester often has a balanced academic load. Another factor could be that by this time, students have adjusted to the medical education system, study patterns, and institutional environment, leading to a more positive outlook.

DREEM domain-specific scores across semesters and gender

In the SPT domain, no significant difference was found across semesters. Hence, no pairwise comparison was run. No significant difference was found across genders. In the SPL domain, a significant difference was found across semesters. A pairwise comparison was run. The third semester showed a significantly higher score than all four other semesters (p < 0.001, in all four individual pairwise tests). No significant difference was found across genders. In the SASP domain, a significant difference was found across semesters. A pairwise comparison was run. The third semester showed a significantly higher score than the first, fourth, and ninth semesters (p < 0.05, in all three individual pairwise tests). No significant difference was found across genders. In the SPA domain, a significant difference was found across semesters. A pairwise comparison was run. The third semester showed a significant difference from the first and fourth semesters (p < 0.05 and p < 0.001, respectively). No significant difference was found across genders. In the SSSP domain, no significant difference was found across semesters. Hence, no pairwise comparison was performed. No significant difference was found across genders.

Our students scored the highest in the domain of students’ perception of learning and felt it was positive. Our findings are consistent with those of Ahmed et al. [15].

Active learning strategies, such as case-based discussions, problem-based learning, and small-group teaching, enhance student engagement and understanding, along with students’ academic self-perception [16]. A clear and systematically designed curriculum followed at our institution also helps students grasp concepts effectively, leading to a better learning experience [17].

Students’ perception of teachers is governed by factors such as effective teaching skills; teachers who use clear explanations, structured lessons, and engaging teaching methods contribute to a positive perception [18]. Our students’ perception of teachers also points toward a more positive approach, as faculty who encourage participation, interactive learning, and personalized attention tend to receive higher scores. The majority of the students believed that teachers demonstrate professionalism, ethical behavior, and clinical competence serve as strong role models for students.

Students’ perception of the atmosphere is one domain where we need to address certain concerns. The high cost of medical education makes academic failures a significant loss of resources for both society and individuals [19,20].

If students feel that teachers are unapproachable or unsupportive, they may struggle to seek guidance, impacting their academic progress. At the institute, we have initiated a Mentor Mentee Program to develop a student-supportive culture to encourage student mentoring programs where faculty members mentor students right from the first semester. This creates student forums or support groups to enhance collaboration. Fair evaluation and assessment policies are adhered to to reduce academic pressure.

Students’ social self-perception was reported as not too bad and can certainly improve. Students’ academic self-perception, encompassing their beliefs and feelings about their abilities and potential in academic settings, is a crucial factor influencing their motivation, engagement, and, ultimately, their academic achievement. Students’ academic self-perception in this study was more on the positive side.

The limitation of this study is that it includes students from a single institution, selected through a convenient sampling method, which restricts the applicability of the findings to other medical institutions. We used the validated DREEM instrument, enrolled a sample sized for adequate statistical power, and applied multivariable analyses with corrections for multiple comparisons. To reduce response bias, surveys were anonymous, and data collectors were blinded to hypotheses. While convenience sampling limits representativeness, it provided a practical foundation for future, broader studies.

## Conclusions

All students had a positive perception of the institutional atmosphere. They found the teaching environment to be relaxed, comfortable, and conducive to concentration. There is a need for a well-structured support system comprising student counselling, stress management initiatives, and a balanced curriculum tailored to the course complexity that is crucial for improving the educational environment. Integrating technology into education can lead to improved learning experiences, enhanced knowledge retention, and increased student engagement by creating immersive and interactive learning environments. Further research is required to explore various solutions for optimizing the educational environment. We suggest conducting this study again in the same institution after implementing reformative measures to address the deficiencies identified in this research.

## Appendices

Annexure 1

**Table 6:** Information sheet for research participants.

Section	Details
Title of research project	Medical students’ perception of educational environment in newly established Institute of National Importance in Eastern Uttar Pradesh: a cross-sectional DREEM questionnaire-based study
Invitation/Introduction	You are invited to take part in this study. Before you decide whether or not to participate, please take some time to understand why it is being done and what it involves. Do not hesitate to contact us if you require any further information or help. (Contact details are provided at the end of this document)
Purpose of the research	The research aims to understand the educational environment at AIIMS Gorakhpur by examining all aspects of students’ learning, social, and interpersonal skill development. The study will assess the current status of the institute in providing an adequate educational environment and offer real-time feedback for further improvements
What do participants have to do	If you wish to take part, please fill out the questionnaire provided along with this information sheet
Confidentiality	Yes. The questionnaire does not require you to provide any personal details such as your name or contact information. All responses will be collected anonymously
Contact for further information	For any queries regarding this research, please contact us at: Email: dramitr@aiimsgorakhpur.in. Thank you for taking the time to read this information. We hope you will help us complete the research project by participating in it

Annexure 2

**Table 7:** ​​​​​​DREEM questionnaire. DREEM: Dundee Ready Education Environment Measure

Question number	Question	Response
1	I am encouraged to participate in class	1. Strongly Disagree, 2. Disagree, 3. Unsure, 4. Agree, 5. Strongly Agree
2	The teachers are knowledgeable	1. Strongly Disagree, 2. Disagree, 3. Unsure, 4. Agree, 5. Strongly Agree
3	There is a good support system for students who get stressed	1. Strongly Disagree, 2. Disagree, 3. Unsure, 4. Agree, 5. Strongly Agree
4	I am too tired to enjoy this course	1. Strongly Disagree, 2. Disagree, 3. Unsure, 4. Agree, 5. Strongly Agree
5	Learning strategies that worked for me before continue to work for me now	1. Strongly Disagree, 2. Disagree, 3. Unsure, 4. Agree, 5. Strongly Agree
6	The teachers are patient with patients	1. Strongly Disagree, 2. Disagree, 3. Unsure, 4. Agree, 5. Strongly Agree
7	The teaching is often stimulating	1. Strongly Disagree, 2. Disagree, 3. Unsure, 4. Agree, 5. Strongly Agree
8	The teachers ridicule the students	1. Strongly Disagree, 2. Disagree, 3. Unsure, 4. Agree, 5. Strongly Agree
9	The teachers are authoritarians	1. Strongly Disagree, 2. Disagree, 3. Unsure, 4. Agree, 5. Strongly Agree
10	I am confident about passing this year	1. Strongly Disagree, 2. Disagree, 3. Unsure, 4. Agree 5. Strongly Agree
11	The atmosphere is relaxed during the ward teaching	1. Strongly Disagree, 2. Disagree, 3. Unsure, 4. Agree, 5. Strongly Agree
12	The school is well timetabled	1. Strongly Disagree, 2. Disagree, 3. Unsure, 4. Agree, 5. Strongly Agree
13	The teaching is student-oriented	1. Strongly Disagree, 2. Disagree, 3. Unsure, 4. Agree, 5. Strongly Agree
14	I am rarely bored in this course	1. Strongly Disagree, 2. Disagree, 3. Unsure, 4. Agree, 5. Strongly Agree
15	I have good friends in this course	1. Strongly Disagree, 2. Disagree, 3. Unsure, 4. Agree, 5. Strongly Agree
16	The teaching is sufficient to develop my confidence	1. Strongly Disagree, 2. Disagree, 3. Unsure, 4. Agree, 5. Strongly Agree
17	Cheating is a problem in this school	1. Strongly Disagree, 2. Disagree, 3. Unsure, 4. Agree, 5. Strongly Agree
18	The teachers have good communication skills with patients	1. Strongly Disagree, 2. Disagree, 3. Unsure, 4. Agree, 5. Strongly Agree
19	The teachers are good at providing feedback to the students	1. Strongly Disagree, 2. Disagree, 3. Unsure, 4. Agree, 5. Strongly Agree
20	The teaching is well-focused	1. Strongly Disagree, 2. Disagree, 3. Unsure, 4. Agree, 5. Strongly Agree
21	I feel I am being well prepared for my profession	1. Strongly Disagree, 2. Disagree, 3. Unsure, 4. Agree, 5. Strongly Agree
22	The teaching is sufficient to develop my confidence	1. Strongly Disagree, 2. Disagree, 3. Unsure, 4. Agree, 5. Strongly Agree
23	The atmosphere is relaxed during the lecture	1. Strongly Disagree, 2. Disagree, 3. Unsure, 4. Agree, 5. Strongly Agree
24	The teaching time is put to good use	1. Strongly Disagree, 2. Disagree, 3. Unsure, 4. Agree, 5. Strongly Agree
25	The teaching overemphasizes factual learning	1. Strongly Disagree, 2. Disagree, 3. Unsure, 4. Agree, 5. Strongly Agree
26	Last year’s work has been a good preparation for this year’s work	1. Strongly Disagree, 2. Disagree, 3. Unsure, 4. Agree, 5. Strongly Agree
27	I am able to memorize all I need	1. Strongly Disagree, 2. Disagree, 3. Unsure, 4. Agree, 5. Strongly Agree
28	I seldom feel lonely	1. Strongly Disagree, 2. Disagree, 3. Unsure, 4. Agree, 5. Strongly Agree
29	My social life is good	1. Strongly Disagree, 2. Disagree, 3. Unsure, 4. Agree 5. Strongly Agree
30	There are opportunities for me to develop interpersonal skills	1. Strongly Disagree, 2. Disagree, 3. Unsure, 4. Agree, 5. Strongly Agree
31	I have learned a lot about empathy in my profession	1. Strongly Disagree, 2. Disagree, 3. Unsure, 4. Agree, 5. Strongly Agree
32	The teachers provide constructive criticism here	1. Strongly Disagree, 2. Disagree, 3. Unsure, 4. Agree, 5. Strongly Agree
33	I feel comfortable in the class socially	1. Strongly Disagree, 2. Disagree, 3. Unsure, 4. Agree, 5. Strongly Agree
34	The atmosphere is relaxed during seminars/tutorials	1. Strongly Disagree, 2. Disagree, 3. Unsure, 4. Agree, 5. Strongly Agree
35	I find the experience disappointing	1. Strongly Disagree, 2. Disagree, 3. Unsure, 4. Agree, 5. Strongly Agree
36	I am able to concentrate well	1. Strongly Disagree, 2. Disagree, 3. Unsure, 4. Agree, 5. Strongly Agree
37	The teachers give clear examples	1. Strongly Disagree, 2. Disagree, 3. Unsure, 4. Agree, 5. Strongly Agree
38	I am clear about the learning objectives of the course	1. Strongly Disagree, 2. Disagree, 3. Unsure, 4. Agree, 5. Strongly Agree
39	The teachers get angry in the class	1. Strongly Disagree, 2. Disagree, 3. Unsure, 4. Agree, 5. Strongly Agree
40	The teachers are well prepared for class	1. Strongly Disagree, 2. Disagree, 3. Unsure, 4. Agree, 5. Strongly Agree
41	My problem-solving skills are being well-developed here	1. Strongly Disagree, 2. Disagree, 3. Unsure, 4. Agree, 5. Strongly Agree
42	The enjoyment outweighs the study of medicine	1. Strongly Disagree, 2. Disagree, 3. Unsure, 4. Agree, 5. Strongly Agree
43	The atmosphere motivates me as a learner	1. Strongly Disagree, 2. Disagree, 3. Unsure, 4. Agree, 5. Strongly Agree
44	The teaching encourages me to be an active learner	1. Strongly Disagree, 2. Disagree, 3. Unsure, 4. Agree, 5. Strongly Agree
45	Much of what I have to learn seems relevant to a career in medicine	1. Strongly Disagree, 2. Disagree, 3. Unsure, 4. Agree, 5. Strongly Agree
46	My accommodation is pleasant	1. Strongly Disagree, 2. Disagree, 3. Unsure, 4. Agree, 5. Strongly Agree
47	Long-term learning is emphasized over short-term learning	1. Strongly Disagree, 2. Disagree, 3. Unsure, 4. Agree, 5. Strongly Agree
48	The teaching is too teacher-centered	1. Strongly Disagree, 2. Disagree, 3. Unsure, 4. Agree, 5. Strongly Agree
49	I feel able to ask the question I want	1. Strongly Disagree, 2. Disagree, 3. Unsure, 4. Agree, 5. Strongly Agree
50	The students irritate the teachers	1. Strongly Disagree, 2. Disagree, 3. Unsure, 4. Agree, 5. Strongly Agree

## References

[1] Khalaf H, Almothafar B, Alhalabi N (2022). Iraqi medical student's perceptions of learning environment following surgical curriculum change. Acta Inform Med.

[2] Thomas P, Kern D, Hughes M, Chen B (2016). Curriculum Development for Medical Education: A Six-Step Approach. https://pure.johnshopkins.edu/en/publications/curriculum-development-for-medical-education-a-six-step-approach.

[3] Ahn D (2020). Current trend of accreditation within medical education. J Educ Eval Health Prof.

[4] Roff S, McAleer S, Ifere OS, Bhattacharya S (2001). A global diagnostic tool for measuring educational environment: comparing Nigeria and Nepal. Med Teach.

[5] Shochet RB, Colbert-Getz JM, Wright SM (2015). The Johns Hopkins learning environment scale: measuring medical students' perceptions of the processes supporting professional formation. Acad Med.

[6] Roff S, McAleer S, Harden RM (1997). Development and validation of the Dundee Ready Education Environment measure (DREEM). Med Teach.

[7] McAleer S, Roff S (2001). A practical guide to using the Dundee Ready Education Environment Measure (DREEM). Curriculum, Environment, Climate, Quality and Change in Medical Education: A Unifying Perspective.

[8] Dunne F, McAleer S, Roff S (2006). Assessment of the undergraduate medical education environment in a large UK medical school. Health Educ J.

[9] Vaughan B, Carter A, Macfarlane C, Morrison T (2014). The DREEM, part 1: measurement of the educational environment in an osteopathy teaching program. BMC Med Educ.

[10] Bavdekar S, Save S, Pillai A, Kasbe AM (2019). DREEM study: students perceptions of learning environment in a medical college in Mumbai, India. J Assoc Physicians India.

[11] Kiran HS, Gowdappa BH (2013). "DREEM" comes true - students' perceptions of educational environment in an Indian medical school. J Postgrad Med.

[12] Awawdeh M, Alosail LA, Alqahtani M (2024). Students' perception of the educational environment at King Saud bin Abdulaziz University for health sciences using DREEM tool. BMC Med Educ.

[13] Kohli V, Dhaliwal U (2013). Medical students' perception of the educational environment in a medical college in India: a cross-sectional study using the Dundee Ready Education Environment questionnaire. J Educ Eval Health Prof.

[14] Pai PG, Menezes V, Srikanth Srikanth, Subramanian AM, Shenoy JP (2014). Medical students' perception of their educational environment. J Clin Diagn Res.

[15] Ahmed Y, Taha MH, Al-Neel S, Gaffar AM (2018). Students' perception of the learning environment and its relation to their study year and performance in Sudan. Int J Med Educ.

[16] Sartania N, Sneddon S, Boyle JG, McQuarrie E, de Koning HP (2022). Increasing collaborative discussion in case-based learning improves student engagement and knowledge acquisition. Med Sci Educ.

[17] Ghasemi MR, Moonaghi HK, Heydari A (2020). Strategies for sustaining and enhancing nursing students' engagement in academic and clinical settings: a narrative review. Korean J Med Educ.

[18] Amerstorfer CM, Freiin von Münster-Kistner C (2021). Student perceptions of academic engagement and student-teacher relationships in problem-based learning. Front Psychol.

[19] Ratnapalan S, Jarvis A (2020). How to identify medical students at risk of academic failure and help them succeed? An interview with a medical educator. Med Sci Educ.

[20] Chou CL, Kalet A, Costa MJ, Cleland J, Winston K (2019). Guidelines: the dos, don'ts and don't knows of remediation in medical education. Perspect Med Educ.

